# Incidence and characteristics of severe exercise-associated collapse at the world’s largest half-marathon

**DOI:** 10.1371/journal.pone.0217465

**Published:** 2019-06-07

**Authors:** H. Lüning, C. Mangelus, E. Carlström, F. Nilson, M. Börjesson

**Affiliations:** 1 Department of Neuroscience and Physiology, Sahlgrenska Academy, University of Gothenburg, Gothenburg, Sweden; 2 Center for Health and Performance, Inst of Food, Health and Nutrition, University of Gothenburg, Gothenburg, Sweden; 3 Inst of Health and Care sciences, Sahlgrenska Academy, University of Gothenburg, Gothenburg, Sweden; 4 Department of Environmental and Life Sciences, Karlstad University, Karlstad, Sweden; 5 Inst of Neuroscience and Physiology, Sahlgrenska Academy, University of Gothenburg, Gothenburg, Sweden; 6 Department of Medicine, Sahlgrenska University Hospital/Ostra, Gothenburg, Sweden; University of Tampere, FINLAND

## Abstract

**Background:**

Whilst many health benefits are associated with regular exercise, medical complications may occur during higher-intensity activities, such as long distance running contests. The most common complication is collapse. However, the incidence and characteristics of these collapses are not very well studied.

**Method:**

This is a retrospective study of severe collapse, defined as a patient in need of advanced medical care after a collapse, during the large Gothenburg’s half marathon, *Göteborgsvarvet*. The study included 230,501 competitors during the study-period of 5 years (2013–2017) with data being collected from medical race tents and using ambulance data. Vital signs, treatment and blood gas samples were noted and analyzed.

**Results:**

The incidence of severe collapse was 1.53 per 1000 starting runners. The average age for patients was 34 years old and no difference in incidence were seen between male and female runners. The typical collapsed runner presented with tachycardia, normal systolic blood pressure, elevated body temperature and metabolic acidosis. The most common medical encounter was exercise-associated collapse.

**Conclusion:**

The incidence of severe collapse was similar to findings in other studies, even though this study was set in different part of the world. Typical characteristics of a collapsed runner were identified providing new information which could be beneficial in the medical planning of larger running competitions and future preventative interventions. Importantly, life threatening conditions seem uncommon; no case of hyponatremia and only two cases of hypoglycemia were seen.

## Introduction

Endurance running has increased considerably in popularity in recent years. In 2013, there were over 50 million recreational runners in the USA and over 18 million had finished an official running race [1]. On a population level, this is exceedingly positive given that regular exercise has been associated with reduced morbidity and mortality, for example having positive effects on hypertension, diabetes mellitus, depression, sarcopenia, etc, acting as both primary and secondary prevention [2, 3, 4, 5, 6]. A recent review also showed runners to have a 25–40% reduced risk of premature death, living approximately three years longer than non-runners [7]. Systematic reviews also show great health benefits, such as lower incidence of obesity, depression and improved bone density, for young and healthy populations [8, 9].

However, high intensity activities, such as endurance running competitions, are also associated with medical complications of different degrees [10]. Although deaths are relatively uncommon [11, 12], a 12-year retrospective study from the Twin Cities Marathon, Minnesota, showed an overall incidence of medical complications of 25.3/1000 finishers, of which 2.53/1000 were classified as severe injuries and illnesses [13]. Another study from South Africa (SAFER study I) showed an overall incidence of 8.3 medical complications/1000 starters, with 0.56/1000 being classified as severe medical complications [10]. In terms of risk factors for medical complications, inexperienced runners in long distance races [14, 15] and older female runners in half-marathons have been shown to be at highest risk [16]. In a study from Singapore half-marathon, Tan et al showed that young runners as well as female runners were at a higher risk of needing medical care [17]. The risk of the sudden cardiac arrests during marathon running, however, is generally low (approximated 1/50 000 participants) [18].

One of the main obstacles for achieving detailed data on different medical complications and especially when comparing risks at different races, has been the lack of a universal definition of a medical complication. At present, the most common medical complication, in available studies, is exercise-associated collapses (EAC), comprising 59% of all medical complications at the Twin Cities Marathon, followed by minor musculoskeletal and skin problems (38%) [13]. The etiology of EAC is multifactorial but is currently hypothesised to be due to the result of transient postural hypotension and impaired baroreflexes after the individual has stopped running [19]. Although EAC is the most common cause of collapses, other causes with similar initial symptoms, such as exertional heat stroke and severe hyponatremia [20], need to be ruled out. These diagnoses are distinguished through clinical presentations, vital signs and blood gas samples to correctly treat and determine these diagnoses at endurance races. EAC may be of different severity with many patients showing a fast recovery while a subset of patients are in need of advanced medical care.

The exact incidence of collapse and medical complications during endurance races in Europe has not been previously explored. Also, the characteristics of collapsed runners, in need of advanced medical care, have not previously been studied in detail. Little is known regarding collapsed runners in terms of blood gas sample values for example electrolytes, blood sugar levels and lactate. Furthermore, little is described regarding vital signs such as blood pressure, pulse and body temperatures. The aim of the present study is therefore to study the incidence of severe collapse during the world’s largest half-marathon race, Gothenburg half-marathon, and to describe these collapses in terms of vital parameters and blood gas values, to provide a more complete clinical picture of the collapsed runner.

## Methods

The current study is a 5-year (2013–2017) retrospective cohort study of collapsed runners during Gothenburg’s half-marathon.

### Ethics

This study was approved by Gothenburg’s Ethical Review Board, ref number 003-17. The patient records were not fully anonymized before accessing the data. When transcribed electronically, all personal data was redacted. The access to data without patients’ consent were approved by the ethics committee.

### Study population

The study includes all starting runners of Gothenburg’s half-marathon during 5 years, 2013–2017. The half-marathon has been in operation for 35 years and is the largest half-marathon race in the world, with around 62,000 entries every year. The race is staged annually in the central parts of Gothenburg, the second largest city of Sweden, in the middle of May [21]. In total, 230,501 starting runners took part in the race during the 5 years, thereby comprising the study population.

Data was retrospectively collected regarding all severe medical complications, i.e. runners in need of advanced medical care at medical tents or in ambulances. The data was derived from medical records (in writing), created for each collapsed runner. This data was obtained from medical stations along the track and from the ambulance registry (AmbuLink) for the runners who were picked-up by an ambulance.

The medical organization of the event is divided into two different types of teams. The first type of team treats minor injuries and complications, such as abrasions and minor musculoskeletal problems. Data from these medical complications were not available and are therefore not included in this study. The second type of medical team, comprised of experienced anaesthesiologists and nurses, treats more serious medical complications. These teams are located in one main medical tent, positioned at the finish line, and in two smaller tents, stationed along the track. The medical organisation also collaborates with the local hospital, Sahlgrenska University Hospital. As part of this collaboration, ambulances are dispatched for pick-ups along the course when needed and treatment can be given at any of the local hospitals.

Runners with mild injuries and complications are generally treated on-site along the track and are not included in this study. Only collapsed runners, deemed in need of advanced medical care, admitted to the medical tents or picked up by ambulances, are included in this study, i.e. here defined as “severe collapses”. Specifically, runners who were admitted to the medical tents being un-responsive and/ or who did not recover after a first medical assessment (e.g. oral fluids). Thus, collapsed runners, studied in the present paper, includes only the category of runners having severe symptoms where initial treatment had failed and who were deemed in need of more advanced medical care. This must be taken into consideration when calculating the incidence rate of severe medical encounters.

### Sample collection

Clinical data was documented on a medical record sheet, created for each patient admitted to the medical tent. This sheet included the starting number, arrival time, date of birth, vital signs (blood pressure, pulse, temperature) clinical symptoms and the treatment given. The medical records were later assembled and transcribed electronically with the personal information redacted. All available data was included. Some medical sheets lacked data, for example data on blood pressure. For this reason, the “n number” can vary between different parameters. In addition to the medical record sheets, data from the ambulance data journal system AmbuLink was collected for the runners who were picked-up by an ambulance and in need of advanced medical care (not treated on-site).

Venous blood gas samples (including pH, pCO2, pO2, Hb, sO2, COHb, MetHb, K+, Na+, Ca2+, Cl-, Glu, Lac) were taken from all runners admitted to the medical tent who showed symptoms where an imbalance of electrolytes and hypoglycaemia needed to be ruled out. The decision whether this was necessary was made on-site by the attending physician based on clinical observations, mainly altered level of consciousness. The blood was analysed immediately, using an on-site blood gas analyser (Triolab), and the results were printed and stored. If the patient was tested multiple times, only data from the initial blood sample was used for analysis in this study. During different years, different machines were used for sample analysis with different analytic setups. Therefore, the “n” numbers vary for some specific blood gas parameters between different years.

### Statistical analysis

Statistical analyses were made using SPSS (IBM 2016).

## Results

### Incidence of collapses

During the study period, a total of 356 runners with severe collapse, in need of advanced medical care, in the medical tents and/or by ambulance transport, were identified amongst 230,501 participants. The incidence was 1.53 per 1000 starting runner varied between 1.19 and 2.21 during the study period (Table 1).

**Table 1:** Vital parameters of collapsed runners in need of advanced medical care.

	N	Minimum	Maximum	Mean	Standard deviation	Low (%)	Normal (%)	High (%)
Body temperature (°C)	218	33.9	41.6	38.2	1.6	2	38	60
Systolic blood pressure (mmHg)	201	55	180	110	21.7	11	77	12
Diastolic blood pressure (mmHg)	168	30	110	58	14.7	47	49	4
Pulse (bpm)	165	60	190	112	21.2	0	21	79

In the medical tents, a total of 276 collapsed runners were treated of which 66% were male and 33% female. This equals the ration of starting runners where 2/3 were male and 1/3 female. The average age was 34 years old (range 17–77). In addition, there was a total of 80 ambulance pick-ups to hospital, due to collapses along the track, averaging 16 per year (range 8–35) (Table 1).

### Characteristics of collapsed runners

All treated patients were collapsed runners. Two patients were treated with IV glucose due to hypoglycemia. Eight runners needed IV diazepam for severe muscle cramps. No case of epileptic cramps was seen. All other collapses were diagnosed as EAC with different accompanying symptoms such as hyperthermia, dehydration, hypotension, disorientation etc. No collapsed runner was observed in the medical stations with chest pain. In the few cases with runners presenting with chest pain, the runners were clinically stable and not collapsed. The runners were immediately sent to hospital and without receiving medical care at the race tent. Thus, no patient with chest pain is included in this study. However, a total of 5 sudden cardiac arrests (SCA) were seen with an incidence of 2.14/100.000 starting runners (Table 1).

Amongst the runners with severe collapse, a majority were seen with elevated body temperature and 18% with hyperpyrexia (body temperatures over 40C). (Table 2). Most collapsed runners showed a normal systolic blood pressure (SBP) with only 11% having a low systolic blood pressure <90mmHg (Table 2). Meanwhile, almost half of the runners showed a low diastolic blood pressure (DBP) (Table 2). Most collapsed runners (79%) showed tachycardia HR >100. None were bradycardic (Table 2).

**Table 2:** Absolute numbers and incidence rates during different study years.

Year	Number of registrations	Starting runners	Number of collapses	Ambulance pickups	Total number of collapses	Injuries/1000 starting runners	Cardiac arrests
2013	64169	46955	69	35	104	2.21	0
2014	64288	48202	59	17	76	1.57	3
2015	64325	47013	55	8	63	1.34	1
2016	64490	45901	51	11	62	1.35	1
2017	60271	42891	42	9	51	1.19	0

### Venous blood gas samples

Venous blood gas samples were derived from a total of 150 patients, i.e. 54% of all patients treated in the medical tents. No blood gas samples were available from the ambulance pick-ups. Venous blood gas samples from collapsed runners showed low (<7.32) pH in 38% of the samples (Table 3). Most (93%) showed elevated lactate levels (Table 3). No samples showed hyponatremia (Na <135mmol/L), however, 63% had elevated sodium levels >145mmol/L (Table 3). Most of the samples showed normal potassium levels and those with elevated potassium levels were normalized when rechecked later. Only 2 samples (1.3%) showed hypoglycaemia (glucose levels <3mmol/L) (Table 3).

**Table 3:** Blood gas samples of collapsed runners in need of advanced medical care.

Measure	N	Mean	Median	sd	Min	Max	Low (%)	Normal (%)	High (%)
pH	150	7.34	7.37	0.12	6.97	7.63	38	36	26
pCO_2_ (kPa)	150	5.07	4.97	1.30	2.10	9.66	63	24	13
pO_2_ (kPa)	146	5.67	5.71	1.95	1.74	13.5	23	21	56
Hb (g/L)	150	154	157	14	108	190	N/A	N/A	N/A
sO_2_ (%)	150	66	70	23	5	98	57	43	N/A
FCOHb (%)	117	0.54	0.6	0.14	0.1	0.8	N/A	100	0
FMetHb (%)	118	0.76	0.75	0.26	0.1	1.4	N/A	100	0
K+ (mmol/L)	150	4.4	4.4	0.64	3.0	7.7	4	80	16
Na+ (mmol/L)	150	146	147	3	136	155	0	37	63
Ca2+ (mmol/L)	150	1.18	1.18	0.05	1.02	1.34	22	77	1
Cl- (mmol/L)	147	106	106	3	98	117	0	86	14
Glucose (mmol/L)	149	5.8	5.5	1.6	2.8	12.7	2	97	1
Lactate (mmol/L)	147	7.7	5.8	5.4	0.9	25.0	N/A	7	93
BE (mmol/L)	119	-4.6	-3.3	5.5	-20.0	6.1	53	44	3
HCO3- (mmol/L)	119	20.4	21.3	3.9	10.8	27.7	51	46	3

### Treatment

Before being admitted to the medical tent, patients were assessed by medical personnel who walked with them outside the tent and gave them oral fluids. The typical treatment for severe EAC is rest in a Trendelenburg position, oral and IV fluids, and observation. A majority (70%) of the patients received IV fluids and 4% IV medications (glucose for hypoglycemia and diazepam for severe muscle cramps). The average treatment time was approximately 1 hour. All patients could be discharged from the medical tents without any need of further medical care except for the patients who arrived late to the medical tent and needed longer recovery time after the medical tent closed.

## Discussion

The incidence of severe collapse in this present study was found to be 1.53 per 1000 starting runners during the study period between 2013 and 2017. Previous studies, also including less severe collapses, have found an incidence of 0.51 [10] and 2.53 per 1000 finishing runners [13], but studies in this field are few. Therefore, this study adds new important information on the incidence of severe collapse in a relatively cool climate. In terms of incidence of collapse, a number of race-related factors, such as daytime air temperature, track conditions, participants’ physical and psychological status, and delays during the event [22, 23, 24, 25, 26] could explain variations between different races in different countries as well as variations of incidence between different years in the same race, aspects that needs further studies.

In this study, there was a 2:1 male/female ratio of patients treated in the medical tents. However, given that there is also a 2:1 ration male/female of starting runners, the risk of severe collapse was similar between males and females. This is in contrast to previous studies that have reported higher overall incidence of medical complications for female runners [16, 17]. However, it should be noted that whilst there is an overall 2:1 male/female ratio at Gothenburg half-marathon, this ratio varies considerably between different age groups. Therefore, this issue needs to be studied further in order to investigate whether the present race has a higher female participation rate than other races, thereby possibly showing a more correct picture of female runners.

When comparing the results from this study to previous studies, several aspects are important to bear in mind. Given the lack of a common definition of medical complications, as well as different methods of data collection, different studies are difficult to correctly compare. For example, in the Twin Cities Marathon study, all injuries and collapses were registered, whilst in this study, only runners suffering from severe collapse and in need of advanced medical care were included. Thereby, it is unsurprising that incidence rates were higher in Roberts study [13].

Importantly, the limitation of merely including severe collapses, is partly a consequence of the medical procedure consisting of an initial assessment and treatment, used in Gothenburg, to determine which runners need advanced medical care. According to the on-site medical personnel, this procedure, including oral fluids and assisted walking, greatly reduces the number of people needing to be further treated in the medical tents and may, therefore, decrease the incidence of severe complications. Assisted walking is a treatment were medical personnel supports and walks the collapsed runner outside the medical tent. This method is hypothesized to support the circulation by activating the lower muscle pump and recirculates pooled blood in the lower extremities which is a main component in EAC [19]. This Gothenburg model has not been described elsewhere in the literature and needs to be studied further.

As was shown in the results, the typical collapsed runner, needing further assistance outside of standard care, was presented with an elevated body temperature, normal SBP, normal or depressed DPB and tachycardia, symptoms that all can contribute to EAC [19]. Many were also seen with hypernatremia indicating dehydration. To the best of our knowledge, this data has not previously been described in more detail in half-marathon runners. One of the main reasons for taking a venous blood gas sample is to rule out life-threatening differential diagnoses for the collapsed runners. In this setting, hyponatremia and hypoglycemia are the main diagnoses to be ruled out. Of all samples in this study, no patients were found with hyponatremia and only two with hypoglycemia. Given that we studied the most severe of the collapsed runners in a large multiple-year sample, i.e. those deemed in need of advanced medical care, it is important to note that the majority had no electrolyte disturbances.

Finally, an important result was the incidence of SCA (2.14/100,000) which was considerably lower than figures shown in international meta-studies (5/50,000) [18]. The causes behind these low incidence rates need to be studied further though could help to explain the recently shown low rates of mortality at Swedish endurance races [27].

### Strength, limitations and future implications

One of the strengths in this study is the novelty of the data. Blood gas samples and vital parameters have not, to our knowledge, been studied in a similar setting. Also, the study was conducted using a large study population during a considerable time period.

The primary limitation of this study is the issue of definitions and therefore the ability to compare the results with other studies. The incidence presented in this study represents severe collapsed runners in need of advanced medical care. The inclusion criteria are narrow but the etiology is broad and could be multifactorial. In future studies, a common and more specific definition is needed in order to make proper comparisons between different studies. This could make pooling of data and thus, international comparisons, possible. With a better knowledge of the etiology of severe collapses during long distance running competitions, better prevention and treatment can be achieved.

A second limitation is that this study lacks data regarding specific diagnoses, mainly as a result of limited data. For example, for practical reasons the body temperature was measured superficially. Therefore, no conclusions regarding incidence of heatstroke can be drawn from our data since no core (rectal) temperatures were measured. Furthermore, it is hard to stratify EAC into specific diagnoses since it composes multiple contributing diagnoses. A typical runner with EAC presented with multiple diagnoses such as heat illness, hypo tension, disorientation, dehydration etc. More detailed data and better medical history for each runner might make this possible in the future.

From a practical perspective, the results from this study can be used for improved planning of endurance races using data regarding severe collapses and the need for specialized health care. With better knowledge of incidence numbers rates and characteristics of medical complications, improved prevention is achievable in the future.

## Supporting information

S1 Dataset Excel data of collapsed runners. (XLSX)
